# Enormous Tumor Developed in the Substance of the Womb

**Published:** 1844-09

**Authors:** 


### SELECTIONS

FROM

### AMERICAN AND FOREIGN JOURNALS.

---

*Enormous Tumor developed in the substance of the Womb.*—We find an account of this extraordinary case in our Spanish contemporary, published at Havana. The subject of it, Isabel Vasquez, was about 40 years of age, unmarried, of nervous temperament, good constitution, correct habits, and a character at times vehement and irascible. In 1831 she contracted a *sub-inflammation* of the womb, which was only partially subdued by the treatment instituted; a relapse occurred before and after each menstrual period. In 1832 and '33, from the elevation, tension, and hardness of the abdomen, an ovaritis was suspected, and iodine was freely used externally and internally. At this time the fears of the reigning epidemic led to the neglect of all plans of cure, and the disease made rapid progress. The abdomen became more tense, the menstrual flux more scanty, the patient was distressed by a feeling of constriction; there were dull pains in the lumbar region, which augmented and finally simulated lumbago; walking was difficult; and after this epoch, erysipelatous affections occurred in the vulva, greater labia, the perineal and hypogastric regions, three or four days before and during the menstrual period; these terminated before or after the appearance of the menses, leaving the skin in a healthy state, except the distension it suffered in consequence of the greater elevation of the tumor. Externally the latter was regular, although a little inclined to the right side of the right iliac fossa, owing perhaps to the habit of inclining the body to that side. At this time also, perhaps in consequence of the weight of the tumor, there was a curvature of the vertebral column, which had almost reached its maximum at the time of the patient's death.

Until 1836 and '37, the disease was somewhat stationary, which was attributed to the use of certain mineral waters, and baths, especially those of Marianao, Cotilla, Obispo, &c. After 1840, the growth of the tumor continued, and the symptoms depending on it increased. Nutrition, which had hitherto been healthy, began to suffer derangement; notwithstanding the patient's appetite was good, the flesh became flabby, and the body emaciated; respiration was slow and difficult, and the want of duly oxygenized blood, contributed further to these alterations. The urinary and other secretions did not suffer any derangement, and were easily excited by the proper remedies. The emaciation, however, was by no means in relation to the enormous size of the tumor.

Menstruation continued through the whole course of her illness, until the month preceding death. Yet the vagina, as shown by the autopsy, was exceedingly contracted, scarcely admitting the extremity of a common sound. This obstruction of the vagina was apparently the cause of the scanty monthly flow, and also of the greater development and increase of the tumor.

The immediate cause of death was a violent and rapid peritonitis, lasting 48 hours only, and consequent upon a rupture of the peritoneum. The rupture occurred during an involuntary and somewhat exaggerated movement on the part of the patient. She felt, at the time, as if *something* had given way; this was soon followed by the most acute pain, by anxiety, fever, coldness, and all the precursory symptoms of death.

We give the narrator's account of the autopsy:

*External Appearance.*—The skin was attenuated, stretched, and lustreless; the features marked; the venous system completely engorged with blood. The vertebral column presented a deviation to the right; the chest, in its inferior third, and the entire epigastric region were distended; the abdomen was so much enlarged that, at its most elevated part, the middle third of the linea alba, it measured *two yards and twenty-seven inches* in circumference, and, from the xiphoid cartilage to the symphisis pubis, *one yard and a third* in length. * * * Beneath the umbilicus were two purple spots, one of which occupied almost the entire sub-pubic region, the other, smaller in extent, occupied the right iliac region; the body presented nothing else worthy of remark, except the emission of caloric from the abdomen, which offered a striking contrast to the icy coldness of death.

*Internal Appearance.*—The abdomen being opened in the whole extent of the *linea alba*, the skin was observed to be attenuated towards the superior part, whilst in the middle and below there was a thickening of more than two inches. The epigastric vein was dilated to twice its natural size and gorged with blood; its ramifications, and the whole venous system, were strongly injected, although the arterial vessels presented the normal calibre. The external and internal oblique muscles, in consequence of the increased length of the abdomen, were greatly attenuated and had almost lost their natural color towards the inferior part, at which point they were separated from the cellular covering uniting them anteriorly to the abdomen, and adhered behind to the peritoneum; the upper parts of these muscles were attenuated, but retained the natural color.

The peritoneum adhered strongly on one side to these muscles, and on the other to the body of the tumor. At this point a *want of continuity* was observed in the membrane; it did not invest the viscera which were found in the epigastric and hypochondriac regions, but, in consequence of the *laceration*, had retracted, leaving them exposed and abandoning its natural relations; it was considerably injected with blood, and had acquired unnatural density and consistence.

All the viscera pertaining to the digestive apparatus had lost their proper position: the stomach, the liver, the intestines, and the pancreas, were displaced by the tumor. The first was flattened at its rhomboidal extremity or cul-de-sac; it was healthy, but inclined towards the right and to the lower part of the right hypochondrium: the liver occupied both hypochondriac regions, and was double the natural size; the slightest pressure sufficed to rupture it: the gall bladder was much dilated, and contained a large quantity of bile: the pancreas and spleen, like the liver, was gorged with blood and brittle: the intestines, pushed towards the lower part of the hypochondriac regions, and inclined to the left, scarcely appeared like the convolutions in those regions; they exhibited spots of phlogosis, and the rectum, at its middle and lower part, had almost lost its cylindrical form, owing to the pressure of the tumor.

The lower half of the abdomen and nearly all the superior was occupied by the tumor, which sprang from the body of the uterus, and affected the same conoidal form. Strongly adherent at the inferior and anterior part to the peritoneum and the muscles of the lower belly, and inclined towards the right, it was free in the superior anterior, posterior inferior, and posterior superior portion. One of the ovaries was completely atrophied, the other was hypertrophied. The Fallopian tubes exhibited no particular lesion, neither did the cavity of the uterus, which not only preserved its original form and dimensions, but the internal membrane was of the natural color, healthy, and lubricated by the fluid natural to it.

The vagina and mouth of the womb were altered in texture and form. The parieties of the former were thickened, and its cavity, as already stated, so narrow as scarcely to admit a common sound; whilst the *os tincæ* was confounded with the body of the womb, and affected externally by the degeneration which formed the base of the tumor. The round ligaments were thickened and distended, indicating the constant struggle they had maintained with the tumor; the left was the more elongated in consequence of the inclination of the tumor towards the opposite side.

The dimensions, thickness, and weight of the immense mass, at the time of extraction, were as follows:

| | |
|---|---|
| Length, - - - - - - - | $1\frac{1}{4}$ yards. |
| Circumference, - - - - - - | $2\frac{2}{3}$ " |
| Thickness and diameter, - - - | $\frac{3}{4}$ " |
| Weight, - - - - - - - | 63 lbs. (avdp.) |

It was of a lardaceous nature, and was confounded with the white and fibrous covering of the uterus. The circular and longitudinal fibres of the latter were considerably enlarged, the uterine veins and arteries were enlarged and thickened, and more dense than natural; they were engorged with blood, but no vestige of mortification was observed either in them, or in the tumor, or in the membrane covering it.

Some irregularities were noticed on the posterior surface, near the apex of the tumor. It should be noted that the part of the tumor corresponding to the right iliac region was more developed, owing doubtless to the curvature of the vertebral column, and the habitual position of the patient. There was another small eminence on the posterior and left side which looked like a *cyst*, but on dividing it no fluid escaped.

The urinary apparatus had suffered no disturbance. The bladder, kidneys, and ureters were healthy. The cavity of the small and large pelves presented no particular alteration, save that the pubic and sacro-iliac symphises were developed as in pregnancy.—*Repert. Medico-Habanero.*

